# Tribo‐Charge Induced Wetting (TCW): A New Wettability Control Mechanism for Electric‐Free Droplet Manipulation

**DOI:** 10.1002/advs.202511863

**Published:** 2025-10-29

**Authors:** Yeonwoo Lee, Sung‐Yong Park

**Affiliations:** ^1^ Department of Mechanical Engineering San Diego State University 5500 Campanile drive San Diego CA 92182 USA

**Keywords:** digital microfluidics, droplet manipulation, electrowetting‐on‐dielectric, surface wettability, triboelectricity

## Abstract

Electrowetting‐on‐dielectric (EWOD) has revolutionized digital microfluidics (DMF) by dynamically controlling surface wettability via electric fields. However, its reliance on external power, patterned electrodes, and complex circuitry limits device simplicity and portability. Here, tribo‐charge driven wetting (TCW) is introduced as a fundamentally new, electric‐free mechanism for active wettability control. Unlike voltage‐driven control in EWOD, TCW employs surface charges generated through contact electrification to modulate surface wettability, eliminating the need for electrodes, wiring, and power supplies. Using TCW, we demonstrate a substantial contact angle modulation of Δ*θ* = 44.4°±1.1, achieved solely by a tribo‐actuator with its surface charge density of σ = −12.61 µC m^−^^2^. Comprehensive experimental and numerical analyses reveal that TCW performance scales strongly with actuator area and charge density but is remarkably insensitive to dielectric thickness within a practical range. Leveraging these features, core DMF operations are demonstrated, including droplet transport, merging, and generation from a reservoir, all accomplished without any electric components. Droplet motion reaches speeds up to 40 mm s^−1^, even across non‐planar terrains. Furthermore, fingertip‐controlled droplet manipulation highlights TCW's simplicity, cost‐effectiveness, and user‐interactivity. This work advances fundamentals of charge‐driven wettability control and establishes TCW as an electric‐free paradigm for active droplet control, opening new opportunities for resource‐limited, lab‐on‐a‐chip and point‐of‐care applications.

## Graphical abstract

Tribo‐charge induced wetting (TCW) is introduced as an electric‐free mechanism for dynamic wettability modulation. TCW utilizes surface charges generated through contact electrification to modulate surface wettability via electrostatic induction across a dielectric layer. A complete set of digital microfluidics (DMF) operations, including droplet transport, merging, and generation from a reservoir, is demonstrated without any electric components.

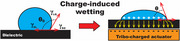

## 1 Introduction

**Figure 1:**
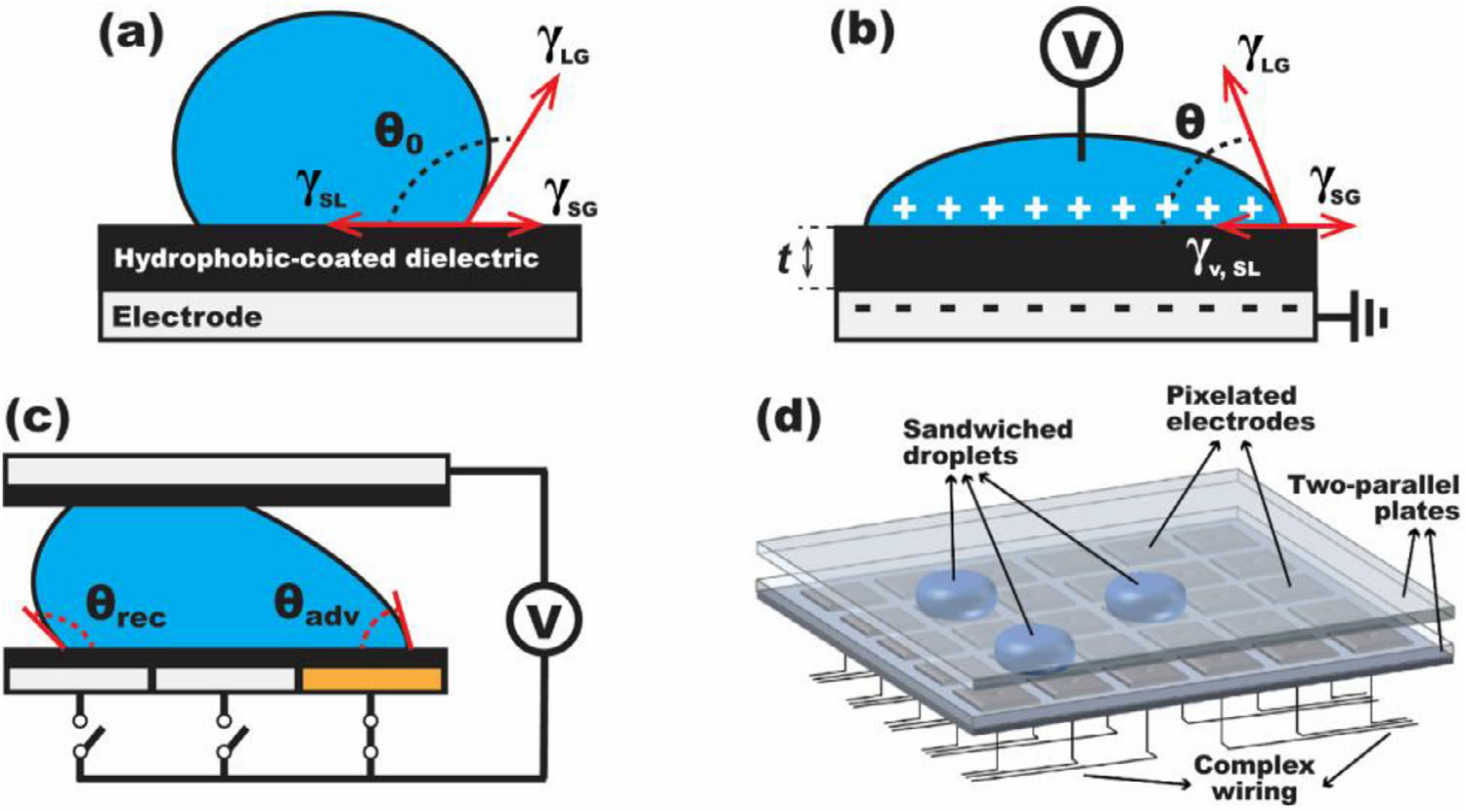
A Working Principle and Limitations of EWOD. (a) A liquid droplet rests on a hydrophobic‐coated dielectric surface, forming an initial contact angle (θ_0_) at equilibrium. (b) When a bias voltage (V) is applied between the droplet and the underlying electrode, electric charges within the droplet are redistributed at the solid‐liquid interface, and a dielectric layer works as a capacitor across which electrostatic energy is stored. This storage energy modifies the surface tension (γ_v, SL_), resulting in the reduction of the contact angle (θ). (c) A droplet is sandwiched between two plates: a patterned electrode array on the bottom and a counter electrode on top. Applying voltage to a specific bottom electrode induces an advancing angle (θ_adv_) at the energized region and a receding angle (θ_rec_) at the trailing edge. This asymmetry generates a pressure gradient that drives droplet motion. (d) EWOD has been developed as a basis of DMF that enables dynamic manipulation of discrete droplets on a 2D arrayed electrode plate. However, EWOD‐driven DMF devices typically encounter the issues of complex electrode fabrication, required power/control circuitry, and vulnerability to dielectric breakdown and electrolysis.

Surface wettability is a fundamental interfacial property governed by the balance of surface tensions at the solid‐liquid‐gas interface.^[^ ^1^, ^2^, ^3^ ^]^ This balance, classically captured by Young's equation,^[^ ^4^ ^]^ defines the equilibrium contact angle (*θ*_0_) on a flat, chemically homogeneous surface (**Figure** **1**a). At the micro/meso‐scales, where surface tension dominates over gravitational and inertial forces, controlling wettability enables precise liquid handling, which is vital for applications in lab‐on‐a‐chip systems, biochemical assays, tunable optics, thermal management, and energy harvesting.^[^ ^5^, ^6^, ^7^, ^8^, ^9^, ^10^ ^]^ Enhanced wettability control in these systems directly translates to improved performance, miniaturization, and operational accuracy.^[^ ^11^, ^12^ ^]^

To meet the demands of dynamic control, researchers have developed active wettability modulation techniques based on external stimuli such as electric fields, light, thermal, or chemical gradients.^[^ ^13^, ^14^, ^15^, ^16^, ^17^ ^]^ Among these, electrowetting (EW) has emerged as a powerful tool for surface energy manipulation.^[^ ^18^, ^19^ ^]^ EW reduces the contact angle of a liquid droplet by applying a bias voltage between the droplet and a solid electrode, thereby storing electrostatic energy across an intervening dielectric layer, as shown in Figure 1b. A resulting contact angle (*θ*) electrically altered by the applied voltage input (*V*) is governed by the Young‐Lippmann equation ^[^ ^20^ ^]^:

(1) $$\;\cos\theta =\cos\theta_{0}+\frac{cV^{2}}{2\gamma_{\mathrm{LG}}}$$

where *c* = *ε_o_ε_r_*/*t* is the specific capacitance, *ε_o_* and *ε_r_* are the vacuum and relative permittivities, *t* is the dielectric thickness, and *γ*_LG_ are the surface tensions at the liquid‐gas interface. The resulting contact angle modulation can be further normalized through the dimensionless EW number (*η*), which is defined as:

(2) $$\eta =\left(\cos\theta -\cos\theta_{0}\right)=\frac{cV^{2}/2}{\gamma_{\mathrm{LG}}}$$

This dimensionless number captures the relative contribution of electrostatic energy to surface tension energy, thus providing a normalized measure of wettability modulation under electric input.

In conventional EW systems, the electric double layer (EDL) serves as a capacitor formed at the interface between the liquid and the solid electrode. However, the ultra‐thin EDL (typically <10 nm) makes it highly prone to dielectric breakdown and electrochemical degradation, limiting its practical use.^[^ ^21^, ^22^ ^]^ To mitigate these issues, electrowetting‐on‐dielectric (EWOD) was developed, replacing the EDL capacitor with a dielectric layer.^[^ ^23^, ^24^, ^25^ ^]^ This EWOD configuration enables improved energy storage, reduced degradation, and larger, reversible contact angle changes on a hydrophobic‐coated surface. A typical EWOD architecture involves a two‐plate sandwich configuration (Figure 1c). The bottom plate comprises an array of individually addressable electrodes coated with a dielectric and hydrophobic layer, while the top plate serves as a common ground or a counter electrode.^[^ ^26^, ^27^ ^]^ Applying a voltage across a selected bottom electrode induces a reduction in contact angle, producing an advancing angle (*θ*_adv_) at the energized site and a receding angle (*θ*_rec_) at the trailing edge. This asymmetry generates a pressure gradient that drives the droplet toward the activated electrode. This voltage‐dependent control enables core digital microfluidic (DMF) operations such as droplet transport, merging, and splitting through sequential activation of patterned electrode arrays.^[^ ^28^ ^]^

Over the past two decades, EWOD‐based DMF systems have transformed microfluidic platforms, leading to commercialization in biomedical and analytical applications.^[^ ^29^, ^30^, ^31^ ^]^ Despite its widespread success, EWOD still persists practical challenges (Figure 1d). First, fabricating the electrode arrays involves complex lithography, multi‐layer printed circuit board (PCB) techniques, or via connections, all of which increase fabrication cost and limit layout flexibility.^[^ ^32^, ^33^ ^]^ Second, the requirement for electric control circuitry and voltage drivers increases system complexity. Lastly, practical EWOD performance is constrained by electrochemical effects, such as dielectric breakdown and bubble formation, leading to device reliability issues. To address these challenges, recent efforts have put on self‐powered EWOD systems using triboelectric nanogenerators (TENGs).^[^ ^34^, ^35^, ^36^ ^]^ In these systems, mechanical motions (e.g., tapping or sliding) are converted by TENGs into electrical energy, which is then routed to EWOD devices to control droplet movement. While eliminating the need for external power supplies, they still require the full electrical infrastructure of traditional EWOD, including patterned electrodes and voltage regulation circuitry.^[^ ^37^, ^38^ ^]^ Some other works utilized corona discharge to make the surface charged for wettability control, but they require high‐voltage power sources (up to 30 kV) to ionize air.^[^ ^39^, ^40^ ^]^ These intrinsic architectural complexities still remain a bottleneck for low‐cost and user‐friendly deployment.

In this work, we introduce tribo‐charge induced wetting (TCW) as a fundamentally distinct and electric‐free mechanism for dynamic wettability modulation. Instead of voltage‐driven wetting control in EWOD, TCW utilizes surface charges generated through contact electrification to modulate the droplet's contact angle via electrostatic induction across a dielectric layer. Thus, TCW eliminates the need for electric components such as 2D patterned electrode arrays, complex wiring, and power sources. Furthermore, the absence of external voltage inputs allows TCW to avoid the risk of dielectric failure (i.e., no bubble formation) that is often observed in conventional EWOD systems, offering an unprecedented level of device simplicity and reliability. We experimentally demonstrate that TCW enables significant contact angle modulation (Δ*θ* = 44.4° ± 1.1), using only a tribo‐charged actuator with surface charge density of σ = −12.61 µC m^−2^. Combined experimental and simulation studies reveal that TCW performance is governed primarily by charge density and actuator size, while being insensitive to dielectric thickness. We also demonstrate a complete set of DMF operations, including droplet transport, merging, and generation from a reservoir, all without any electric components. Furthermore, TCW enables user‐interactive control through fingertip actuation, highlighting its simplicity, accessibility, and intuitive operation. This work not only deepens the fundamental understanding of charge‐induced wetting but also lays the foundation for a new class of low‐cost, energy‐independent, and user‐friendly microfluidic platforms for potential applications in diagnostics, environmental sensing, and mobile healthcare.

## 2 TCW Fundamentals

### 2.1 An Electric‐Free Wettability Control

**Figure 2:**
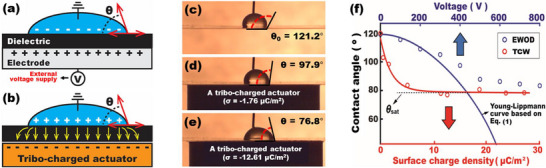
Experimental Demonstration and Comparative Analysis of TCW with EWOD. Schematics of (a) EWOD and (b) TCW mechanisms for surface wettability control. Proof‐of‐concept demonstrations of charge‐driven wettability control at (c) an initial state, (d) σ = −1.76 µC m^−2^, and (e) σ = −12.61 µC m^−2^. (f) Contact angle response of EWOD (blue) and TCW (red) as a function of actuation strength.

Conventional EWOD relies on external power supplies to modulate surface wettability through electric energy stored in a dielectric layer (**Figure** **2**a). In contrast, TCW represents a fundamentally different approach by replacing such voltage‐driven control with charge‐driven wettability modulation. Instead of external voltage sources in EWOD, TCW relies on a tribo‐electrically charged materials as external actuation sources to generate electric fields across the dielectric. This tribo‐charged actuator is simply created by rubbing two dissimilar materials via contact electrification. Depending on their relative electron affinities, these materials exchange electrons upon contact and separation, resulting in the accumulation of surface charges.^[^ ^41^ ^]^ When this tribo‐charged actuator is placed beneath a droplet resting on a dielectric layer, the surface charges redistribute opposite charges within the droplet at the solid‐liquid interface through electrostatic induction, creating a local electric field across the dielectric layer and thus modulating the contact angle (Figure 2b). This TCW mechanism enables electric‐free wettability modulation, without any wiring, voltage regulation, or patterned electrodes that are essential for traditional EWOD systems.

### 2.2 Experimental Validation of TCW Modulation

The triboelectric series presents the rank of materials based on their tendency to gain or lose electrons.^[^ ^42^, ^43^ ^]^ To maximize the charge transfer effect, material pairs from opposite ends of the triboelectric series are selected. In this study, polytetrafluoroethylene (PTFE) and nylon were chosen due to their high electron‐donating and ‐accepting abilities, respectively.^[^ ^44^, ^45^ ^]^ By adjusting a normal frictional force during contact between PTFE and nylon, a negatively charged PTFE actuator was produced, and its surface charge density was variously tuned. Increasing the frictional load improved surface conformity, reducing air gaps and enabling more effective charge transfer.^[^ ^46^, ^47^ ^]^ This simple mechanical tuning allowed us to control the surface charge density up to σ = −55.36 µC m^−2^.

We validated this TCW principle experimentally using a 30 µL water droplet placed on a 25 µm thick PTFE film, which served both as the hydrophobic surface and dielectric layer. Initially, the droplet exhibited its contact angle of *θ*_0_ = 121.2° (Figure 2c). Introducing a lightly charged PTFE actuator (σ = −1.76 µC m^−2^) reduced the contact angle to 97.9° (Figure 2d). Increasing the surface charge density to σ = −12.61 µC m^−2^ further reduced the angle to 76.8°, demonstrating a total angle modulation of Δ*θ* = 44.4° (Figure 2e). Higher charge densities attract a greater number of opposite charges at the solid‐liquid interface. This enhances electric field strength across the dielectric, thus improving contact angle modulation.

In these wetting experiments, the droplet was temporarily grounded to facilitate more efficient charge redistribution at the interface, enhancing the observable contact angle change. However, this ground connection was fully removed in all subsequent droplet manipulation experiments to ensure a truly electric‐free TCW operation (to be discussed in Section 4). It is also noted that the droplet returned back to an original state when the actuator was removed, showing reversibility in angle modulation. These results confirm that TCW enables robust and tunable wettability modulation using only by surface charges, offering a dramatically simplified architecture for electric‐free microfluidic operations.

### 2.3 Comparative Analysis of TCW with EWOD

Despite fundamental differences between EWOD and TCW in energy sourcing, both mechanisms rely on electrostatic modulation of the solid‐liquid interfacial tension and thus share several operational characteristics. In this section, we compare EWOD with TCW through experimental results to highlight the unique advantages of TCW. Figure 2f presents contact angle change as a function of actuation strength for both EWOD (blue) and TCW (red). These results were obtained under matched experimental conditions. A comparative analysis reveals the following:

- Actuation source

In EWOD, angle modulation is governed by an externally applied voltage. In contrast, TCW replaces the electrical input with mechanically generated tribo‐charges. When placing below the droplet, these charges induce electric fields across the dielectric without direct voltage inputs. The degree of contact angle modulation in TCW scales with the surface charge density (σ), that is, higher σ yields a stronger electric field and thus a greater wettability change.

- Contact angle saturation

EWOD exhibits contact angle saturation, the well‐known limitation where further increases in voltage yield negligible contact angle change.^[^ ^48^ ^]^ A similar saturation behavior was observed for TCW, with a limiting angle of approximately *θ*_sat_ = 76.8°. Beyond this point, increases in surface charge density do not produce appreciable changes in droplet shape. This indicates that TCW shares the same fundamental saturation limit, despite operating via a different mechanism.

- No bubble formation

One practical issue of EWOD systems is gas bubble generation caused by electrolytic decomposition due to material imperfections or dielectric breakdown.^[^ ^49^ ^]^ With a 25 µm thick PTFE film as a dielectric layer, we have often observed such failure in EWOD under high voltage applications (≈hundreds of volts). In contrast, TCW inherently avoided this issue due to the absence of external voltage inputs. Even at high σ values (above 40 µC m^−2^), no bubble formation has been experimentally detected in TCW. An additional test on the effect of increased conductivity via the addition of salt in the same water droplet (12% w/w) still showed no bubble formation. Thus, TCW offers a fundamentally safer and more robust platform for handling a broad spectrum of fluids including ionic liquids and biological buffers, without the risk of dielectric failure commonly associated with EWOD.

- Angle modulation efficiency

EWOD exhibits a gradual, voltage‐dependent decrease in contact angle, while TCW responds to charge‐driven angle modulation rapidly and more effectively over its operating range. With the actuator charged to σ = −12.61 µC m^−2^, we achieved a total modulation of ∆*θ* = (*θ*_0_ – *θ*) = 44.4°, without compromising device reliability by material degradation or breakdown.

These findings confirm that TCW replicates the core functionality of EWOD while offering several distinct benefits such as simplified architecture, enhanced electrochemical stability, and independence from electronic components.

### 2.4 TCW Governing Parameters

To gain deeper insight into the governing factors of TCW, we experimentally investigated how material‐related parameters, such as charge stability, actuator size, and dielectric thickness, affect wettability modulation. These studies are crucial for optimizing TCW performance, ensuring long‐term stability, and designing reliable electric‐free DMF systems.

- Surface charge stability

Charge retention over time is essential for reliable and sustainable TCW operation. We monitored charge retention of PTFE actuators in ambient air over 10 min. Interestingly, discharging behavior of the actuator depended on its initial charge density (see Figure S1, Supporting Information). Actuators with high initial charge density (e.g., σ = −41.25 and - 55.36 µC m^−2^) exhibited more than 15% charge loss over 10 min. In contrast, moderate charge densities at σ = −9.11 and - 21.33 µC m^−2^ maintained more than 96.7% of their initial charges. This observation can be attributed to the onset of air breakdown near σ = 27 µC m^−2^.^[^ ^50^ ^]^ Surface charge densities above this onset value trigger ionization of surrounding air molecules, consequently making them conductive locally and accelerating charge leakage. These results suggest a practical upper bound for charge density, beyond which atmospheric effects degrade performance. Fortunately, most TCW operations require surface charges well below this threshold, offering excellent temporal stability in real‐world use.

- Actuator size

**Figure 3:**
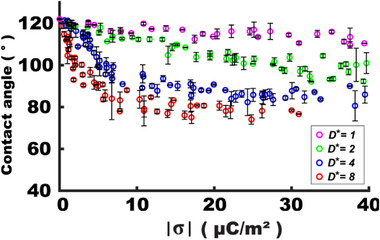
Actuator's size dependency on TCW‐based wettability control. A 30 µL water droplet is loaded on the surface of a 25 µm thick PTFE film. The base contact diameter is 3 mm. It is clearly shown that larger actuators enhance more contact angle modulation. D* denotes the actuator's size (*D*_A_) normalized to the droplet size (*D*_D_) in diameter.

In TCW, the total charge (*Q*) is not only a function of surface charge density (σ) but also of the actuator area (*A*), that is, *Q* = σ*A*. This implies that larger actuators attract a greater number of charges at the interface with stronger electric fields across the dielectric, thereby enhancing wettability modulation. Experimental results in **Figure** **3** show a dramatic size dependence. An actuator size is normalized to the droplet diameter as D* = *D*_A_ / *D*_D_, where *D*_A_ is the actuator diameter and *D*_D_ is the base contact diameter of the droplet. At σ = −13.76 µC m^−2^, the angle change was modest as ∆*θ* = 4.5° for D* = 1. In contrast, with D* = 8, the angle modulation increased dramatically to ∆*θ* = 44.4° even with a slightly lower charge density at σ = −12.61 µC m^−2^. This contrasts with EWOD systems, where angle modulation depends on electrostatic energy per unit area and is not dependent of electrode size, as described in the Young–Lippmann Equation (1). This size‐dependent TCW study will be further discussed with simulation data in Section 3.2.

- Dielectric layer thickness

**Figure 4:**
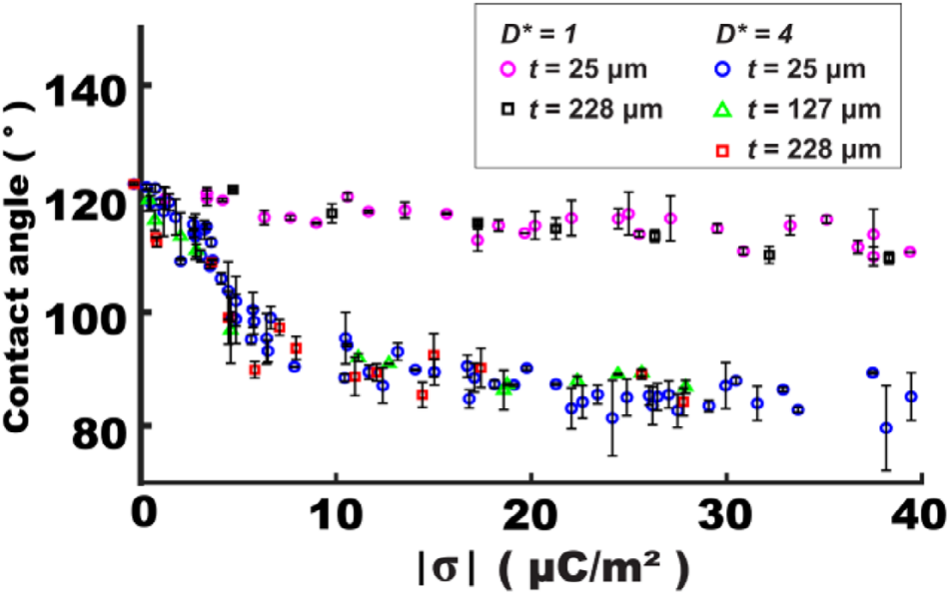
Thickness effect on TCW‐based wettability control. Despite a 9.1‐fold thickness difference (25–228 µm), no considerable effect is observed on angle modulation at both D* = 1 and 4.

In EWOD, a thinner dielectric layer is preferred to enhance wettability modulation by increasing the specific capacitance. However, the minimum thickness is practically limited due to the risk of dielectric breakdown in high‐voltage operations. In contrast, TCW operates without direct electrical bias, thereby eliminating the practical concerns related to breakdown and bubble formation. Experimental results in **Figure** **4** reveal nearly identical contact angle changes across a wide thickness range (25–228 µm) for both the actuator's sizes at D* = 1 and 4. This thickness‐independent TCW modulation will be further discussed using simulation data in Section 3.2.

## 3 Numerical Simulation Study

While the Young‐Lippmann Equation (1) provides an analytical framework for predicting EWOD performance, this direct application is not feasible for TCW. Unlike EWOD, TCW operates without applied voltage, and the electric field arises entirely from surface charges induced via contact electrification. Consequently, direct field strength measurement is impractical, and simple capacitor models ^[^ ^51^ ^]^ fall short in accounting for TCW's geometric and material effects. To address these challenges, we employed three‐dimensional (3D) finite‐element simulations to analyze electric field distributions and induced voltage drops (or effective voltages) across the dielectric layer. These simulations will help elucidate how key design parameters – such as actuator size, charge density, dielectric thickness, and permittivity – affect the strength and spatial distribution of the electric field.

### 3.1 Electric Field and Voltage Distributions

**Figure 5:**
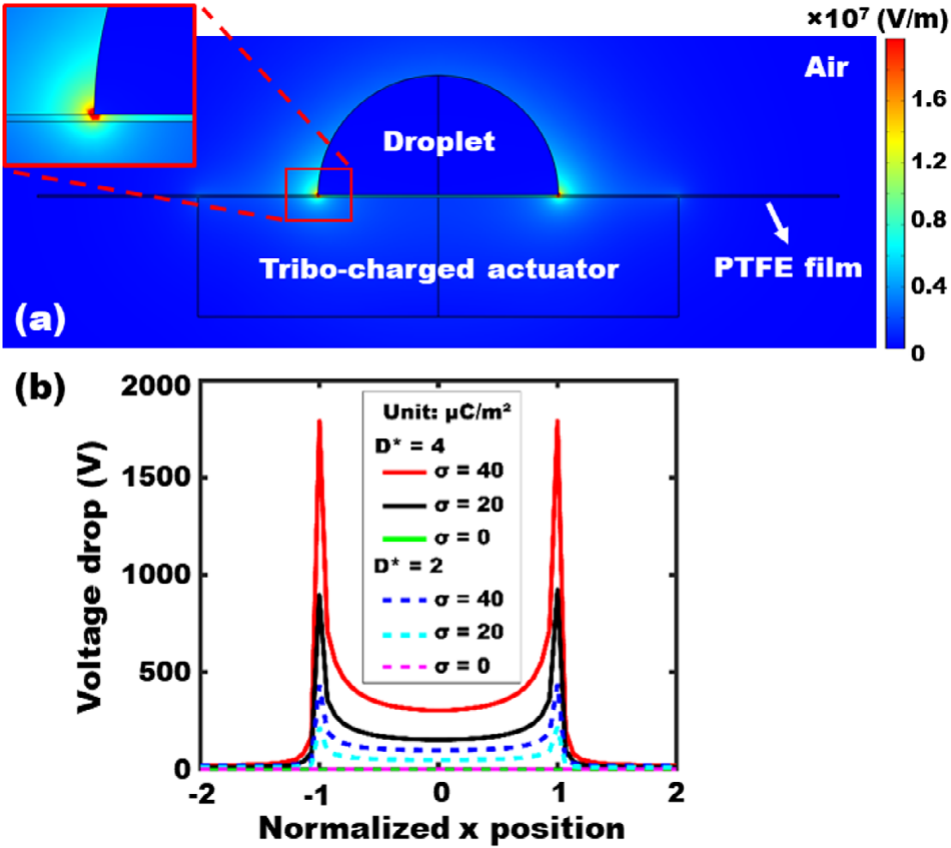
(a) 3D numerical simulation of the electric field distribution created by a circular actuator in a 6 mm diameter positioned below a 3 mm hemispherical droplet sitting on the surface of a 25 µm PTFE dielectric layer. (b) The voltage drop profiles across a dielectric layer are extracted from the simulations and plotted along the lateral cross‐sectional position (*x*‐axis) normalized to the droplet size, while varying the actuator's charge density.

Using COMSOL Multiphysics, we constructed 3D models under the same experimental conditions. The electric field (*E*) was computed by solving Gauss's law; ∇ *• E* = *ρ*/*ε*, where *ρ* is the surface charge density of the actuator and *ε* is the dielectric permittivity of the materials within the simulation domain. **Figure** **5**a shows a representative 3D simulation of the electric field created when a circular actuator is placed below a droplet. Due to fringing effects, the field strength is enhanced at the droplet perimeter (see an enlarged image). Voltage drop profiles across the dielectric layer were then extracted along the lateral cross‐sectional position (*x*‐axis) normalized to the droplet size, and plotted in **Figure** 5b for varying actuator charge densities and sizes (D* = 2 and 4). With higher charge density and larger actuator, a greater number of charges are correspondingly re‐distributed at the solid‐liquid interface and create a stronger electric field, resulting in higher potential distributions across the dielectric layer. These profiles are crucial for calculating the effective voltage experienced by the droplet in TCW and for understanding how geometric and material parameters influence contact angle modulation.

### 3.2 Material Effects on TCW Performance

Using the effective voltages obtained from the 3D simulations, we assessed how key material parameters, such as charge density (σ), actuator size (*A*), dielectric permittivity (*ε*_r_), and dielectric thickness (*t*), influence the wettability modulation in TCW. These effects were analyzed using the dimensionless EW number (*η*), which represents a normalized measure of contact angle modulation, as described in Equation (2).

- Charge density and actuator size

**Figure 6:**
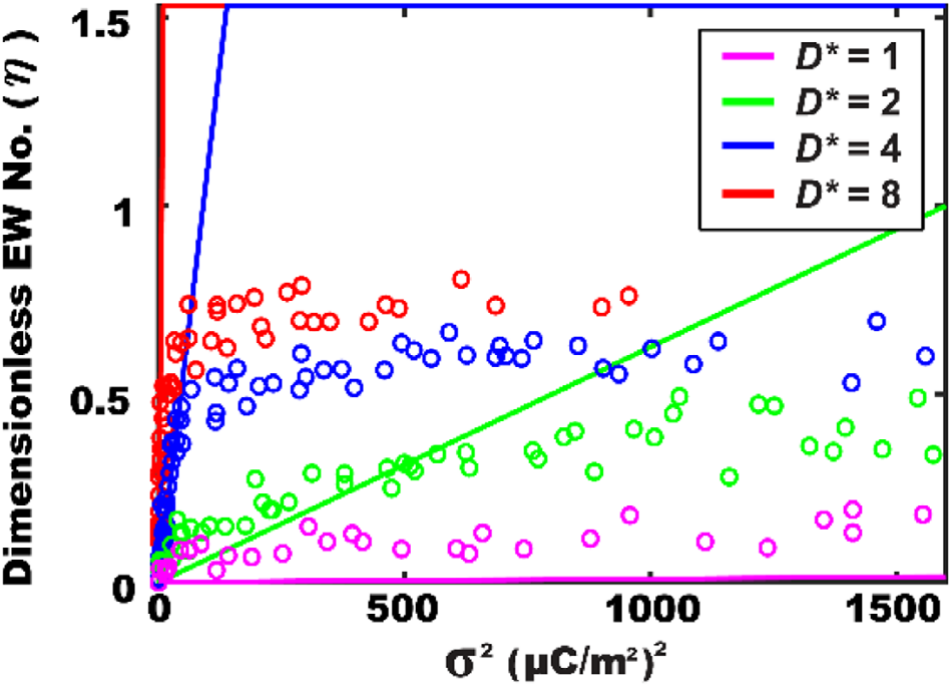
The dimensionless EW number (*η*) as a function of σ^2^. A linearly increasing relationship is clearly observed between *η* and σ^2^. An increasing trend becomes further steeper for a larger size of the actuator. D* denotes the actuator's size normalized to the droplet size in diameter.

**Figure 7:**
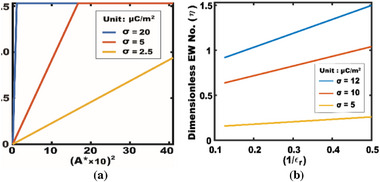
Simulation results of *η* as a function of (a) the square of the actuator area normalized to the droplet area (A*)^2^ and (b) an inverse of a relative permittivity (1/*ε*_r_).

Simulation results in **Figure** **6** show a clear linear relationship between *η* and the square of the charge density, *η* ∝ σ^2^, consistent across different normalized actuator sizes (D*) in diameter. These simulation results are corroborated by experimental data replotted from Figure 3, showing good agreement (except for D* = 1) up to the contact angle saturation regime. Notably, the slope of *η* versus σ^2^ becomes steeper for larger D*. A key insight here is that actuator size directly contributes to wettability modulation in TCW, unlike EWOD, where its performance is based on per unit area. As discussed in Section 2.4, the total charge (i.e., *Q* = σ*A*) matters in TCW, and actuator size becomes an adjustable design parameter for optimizing its performance. Further analysis shown in **Figure** **7**a confirms that *η* scales linearly with the squared normalized actuator area to the droplet base area, that is, (A*)^2^, with a steeper increase by a higher charge density. These findings support that both charge density and actuator area critically influence TCW‐driven contact angle modulation.

- Dielectric permittivity

In conventional EWOD, high‐permittivity dielectrics are preferred to increase the specific capacitance, allowing greater wettability modulation for a given voltage. However, in TCW, the permittivity effect is counterintuitive and inversely correlated with its performance, because the surface charges generate the electric field through electrostatic induction. Simulation results in Figure 7b reveal an inverse relationship between *η* and *ε*_r_, with a steeper slope by a higher charge density. This behavior arises because high‐permittivity materials effectively screen the field induced by surface charges, reducing the effective voltage across the dielectric and thus contact angle modulation. As a result, low‐permittivity materials are advantageous in TCW – a design principle that is opposite to EWOD.

In our experiments, a PTFE film was used as a single layer that provides both dielectric and hydrophobic surface properties. Since this material requirement limits in finding extra dielectric materials, we couldn't have additional data to study the permittivity effect on TCW in experiments.

- Dielectric thickness

**Figure 8:**
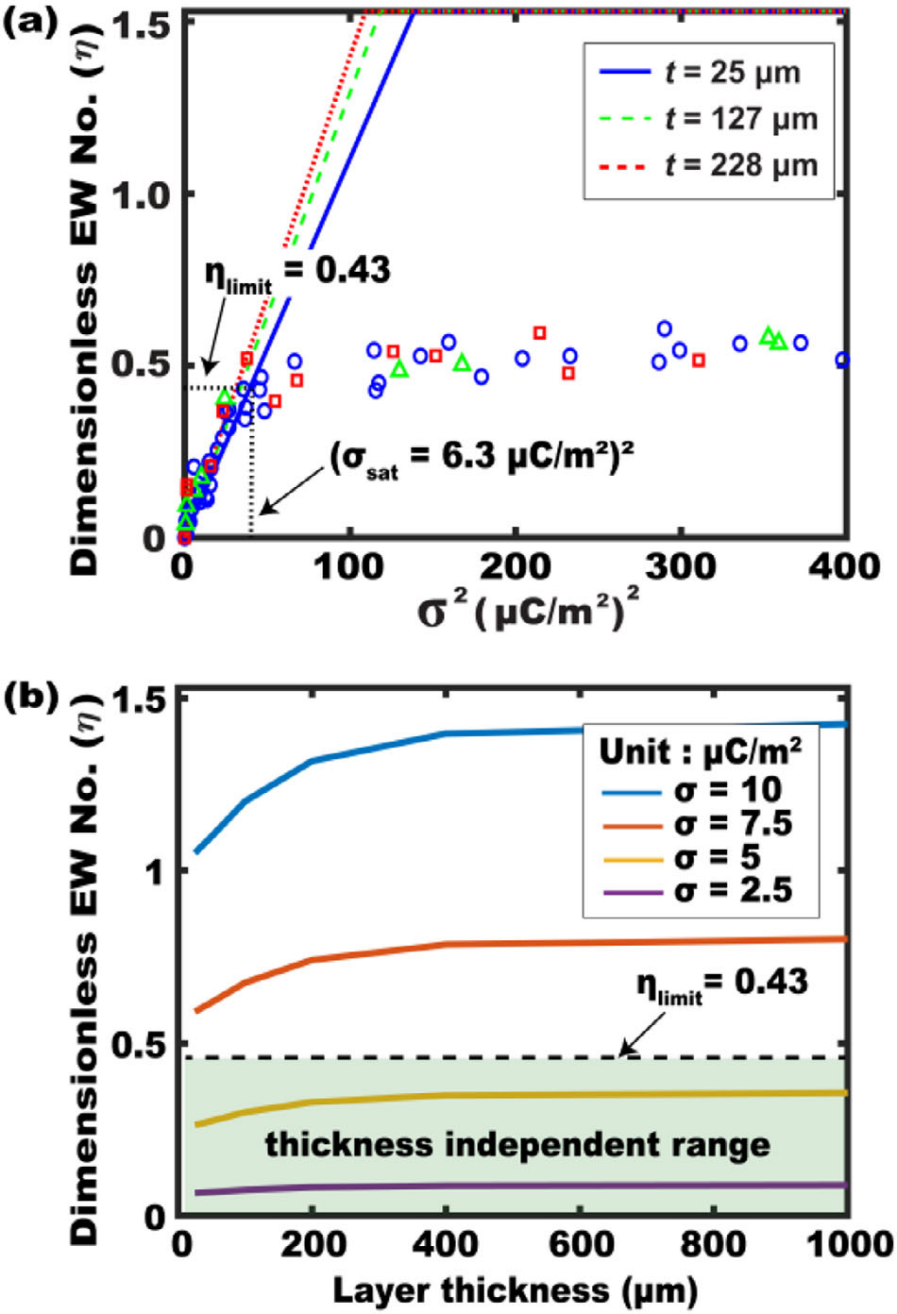
Thickness effect on TCW performance. (a) Both experimental and simulation results are plotted for *η* as a function of σ^2^, while varying the dielectric thickness (25–228 µm) at D* = 4. The simulation results show a slight improvement in *η* for thicker dielectric layers. However, this improvement is minimal and difficult to be detected in experiments before reaching saturation angles. (b) Additional simulation results show *η* varied with layer thickness. When considering a practical working range below the saturation limit at *η*_limit_ = 0.43, we would say that TCW performance is effectively thickness‐independent in its useful operating range.

**Figure** **8**a compares simulation and experimental results (replotted from Figure 4), examining the thickness effect for D* = 4. Simulations show a slight improvement in *η* for a thicker dielectric layer at a given σ. However, this improvement remains minimal, particularly below the saturation limit at *η*_limit_ = 0.43 where the contact angle saturation begins. Even across a 9.1‐fold thickness range (25–228 µm), the enhancement is negligible and difficult to detect experimentally.

Figure 8b shows additional simulation results of *η* varied with dielectric layer thickness. Its variation becomes minimal for thicker layers and lower charge densities. Particularly, considering a practical operation range below the saturation limit at *η*_limit_ = 0.43 (colored green), the *η* variation remains nearly negligible (less than 3° in contact angle changes) even across a 40‐fold thickness range (25–1000 µm). These findings support that dielectric thickness is a relatively insensitive parameter for TCW systems within the practical working range. To generalize this, we further repeated this simulation study for various actuator sizes (see Figure S2, Supporting Information). Across all configurations, *η* nearly plateaus within a practical working range, confirming thickness‐independent TCW performance in its useful operating range.

This nearly thickness‐insensitive behavior in TCW is a unique phenomenon not observed in EWOD systems, where dielectric thickness critically impacts its performance. This TCW feature enables device fabrication on a broad range of substrates – including thick, flexible, or low‐cost films – without sacrificing its performance.

## 4 Experimental Demonstrations of Electric‐Free Droplet Manipulation

Building on the theoretical foundation and simulation insights from previous sections, we now demonstrate the practical capabilities of TCW for fully electric‐free DMF operations. All experiments were conducted without any electrical components, including power supplies, wires, and patterned electrodes. Even the ground connection used in earlier contact angle experiments was fully removed in these demonstrations, while tribo‐charged actuators served as the sole actuation source, enabling completely electric‐free droplet operations. These demonstrations validate TCW not only as a scientific novelty but also as a robust and scalable platform for real‐world microfluidics applications where simplicity, portability, and energy‐independence are paramount.

### 4.1 Core DMF Operations Enabled by TCW

- Continuous droplet transport

**Figure 9:**
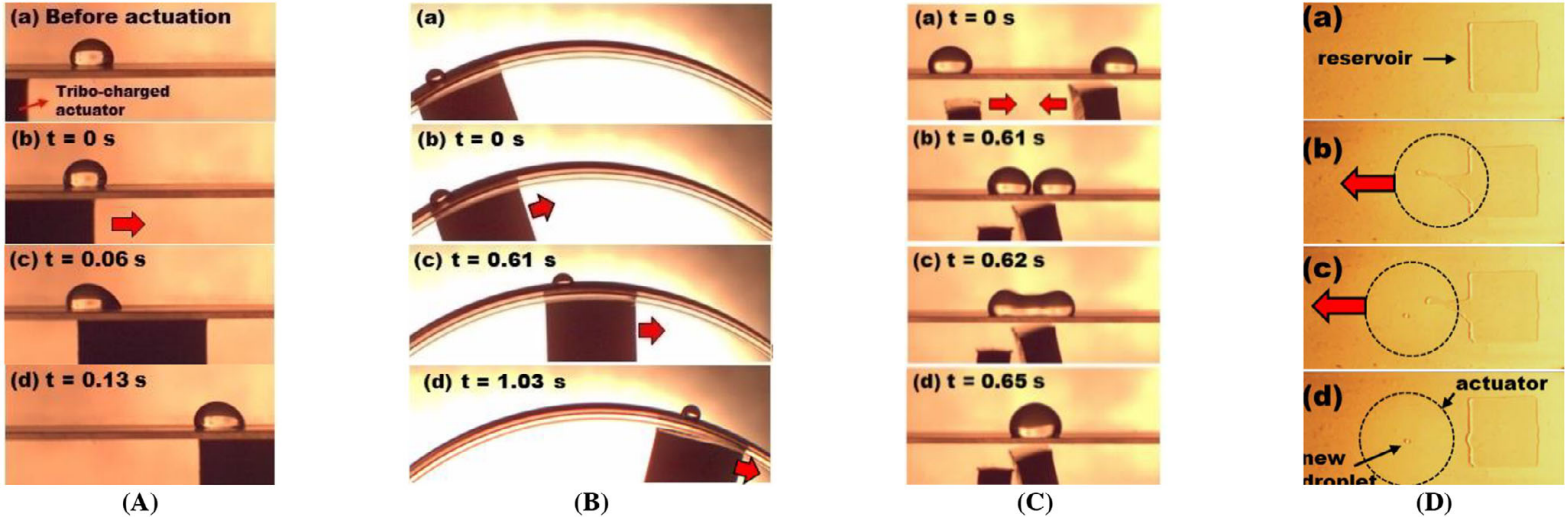
Experimental demonstration of the TCW's capabilities for (A) droplet transportation at a speed of 40 mm s^−1^ on a planar surface, (B) transportation on a non‐planar curved surface, (C) two‐droplet merging, and (D) droplet generation from a reservoir using a circular PTFE actuator. No electric components were equipped for all experiments, demonstrating an electric‐free TCW operation for droplet manipulation.

Core DMF operations have been demonstrated by only using tribo‐charged actuators (Movie S1, Supporting Information). **Figure** **9**A shows video snapshots for demonstrating continuous droplet transport on a planar substrate. A 30 µL water droplet was placed on a 90 µm PTFE film gently covered on top of a 650 µm poly(methyl methacrylate) (PMMA) substrate. No droplet movement was initially observed when a PTFE actuator, charged to σ = −21.44 µC m^−^^2^, was positioned away from the droplet. As the actuator was moved at a speed of 40 mm s^−1^, the droplet was seen to elongate, deform, and follow the actuator's trajectory across the surface. These wetting behaviors for droplet transport are further detailed in Figure S3 (Supporting Information). The experimental results confirm that the charge‐induced electrostatic field effectively creates a surface energy gradient, driving droplet motion, analogous to EWOD, but entirely in an electric‐free manner. This charge‐driven droplet actuation is also well supported by additional simulation study where actuator displacement from the center results in asymmetric voltage drop across the dielectric at the left and right edges of the droplet, which is further discussed in Figure S4 (Supporting Information).

- Transport across a curved surface

To explore TCW's versatility on complex geometries, we also demonstrated droplet actuation over non‐planar surfaces (Figure 9B). A curved terrain was created by wrapping a PTFE film over the 800 µm thick sidewall of a petri dish made of polycarbonate (PC). A 10 µL water droplet was loaded on top of the PTFE surface and held by placing an actuator below. The droplet was successfully transported over this curved surface only using the actuator charged to σ = −25.30 µC m^−^^2^, highlighting TCW's robustness on 3D topographies.

- Droplet merging

Merging is a fundamental operation in DMF used for sample mixing, chemical reactions, or biological assays. As shown in Figure 9C, two 50 µL droplets were placed side‐by‐side on a PTFE‐coated PMMA surface, the same substrate as before. Two separate PTFE actuators, each charged around to σ = −18.72 µC m^−^^2^, approached each droplet from opposite sides, simultaneously to push them toward the center. Upon contact, the droplets coalesced spontaneously, completing the merging process. This process occurred entirely through the mechanical motion of the actuators, demonstrating TCW's ability to support fluidic reactions without electrical input.

- Droplet generation from a reservoir

Droplet creation from a reservoir ‒ essentially droplet cutting ‒ is typically more challenging, as it requires sufficient Laplace pressure to overcome surface tension.^[^ ^28^ ^]^ To demonstrate this, we constructed a confined reservoir between two parallel plates: a lower PTFE‐coated PMMA substrate and an upper glass plate coated with a 490 nm thick Teflon layer, except at a central 9 × 9 mm^2^ hydrophilic zone to anchor the fluid. An 8 µL water droplet was then compressed between these plates, forming a reservoir with a 100 µm gap (Figure 9D). As a circular actuator (12 mm in diameter), charged to σ = −48.99 µC m^−^^2^, was dragged beneath the reservoir. This extracted a liquid neck from the reservoir and pinched off an 18.5 nL daughter droplet, enabling electric‐free droplet creation in the range of nanolitres.

### 4.2 Fingertip‐Driven, User‐Interactive Droplet Control

**Figure 10:**
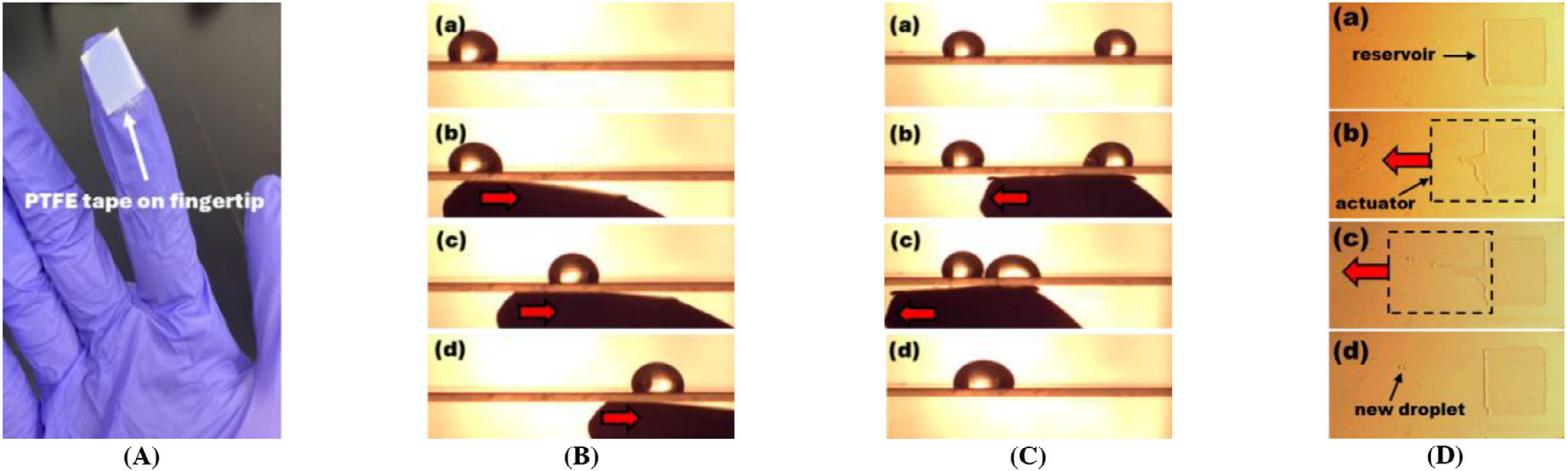
Electric‐free TCW operation by simple fingertip motion. (A) A fingertip actuator was fabricated by attaching a small piece of the PTFE tape (11 × 14 mm^2^ and 254 µm thick) on the edge of the index finger covered by a nitrile glove. Using simple fingertip motion, a variety of dropwise manipulation functions have been demonstrated, including (B) droplet transport, (C) merging, and (D) droplet generation from a reservoir. These experimental results demonstrate simple and electric‐free TCW operation achieved by the user's finger motion, offering a highly accessible, cost‐effective, and versatile platform suitable for diverse DMF applications.

One of TCW's most compelling attributes is its compatibility with intuitive, user‐interactive interfaces. To illustrate this, we fabricated a fingertip actuator by simply adhering a small piece of the PTFE tape (11 × 14 mm^2^, 254 µm thick) to a nitrile‐gloved index finger (**Figure** **10**A). After rubbing it against a nylon board to generate tribo‐charges on its surface, the fingertip was used as a direct, user‐controlled droplet actuator. This intuitive method enabled all the above droplet manipulation functions using simple fingertip motion (Movie S2, Supporting Information). Figure 10B presents video snapshots of continuous transportation of a 30 µL droplet on a planar PTFE‐coated PMMA substrate using only the motion of the fingertip actuator at σ = −14.56 µC m^−2^. Droplet merging was also achieved in a similar manner using the fingertip actuator (Figure 10C). Figure 10D shows an experimental demonstration of droplet creation from the same reservoir setup. Using the fingertip's dragging motion, a 17.6 nL droplet was successfully generated from the reservoir by the fingertip actuator at σ = −9.11 µC m^−2^.

These demonstrations showcase a new paradigm of human‐interactive digital microfluidics, where droplets can be manipulated in real time using only touch, with no electronics, wires, or control software.

### 4.3 Practical Advantages of TCW over Traditional EWOD

The experimental validations described above emphasize several unique practical benefits of TCW, especially in comparison with conventional EWOD platforms.

- Cost‐effectiveness and system simplicity

TCW completely eliminates the need for microfabricated electrode arrays, wiring, external power supplies, and control electronics, typically associated with conventional EWOD systems. The core materials, such as PTFE and nylon, experimentally used in this study, are commercially available, low‐cost, and disposable. Actuators can be quickly prepared by simply rubbing each other and instantly replaceable without requiring surface treatment or post‐processing. This drastically reduces both manufacturing cost and operational complexity, offering a highly simplified platform that is affordable, easy to maintain, and ideal for low‐resource environments.

- Material compatibility and flexibility

TCW offers remarkable material compatibility and design freedom. While a PTFE film serves as the primary hydrophobic contact surface for droplets, a variety of substrate materials have been successfully demonstrated for TCW operation – including PMMA, PTFE, poly(ethylene terephthalate) (PET), PC, polyethylene, polypropylene, polydimethylsiloxane (PDMS), Kapton tape, and Parafilm tape – many of which inherently are flexible, biocompatible, chemically inert, or transparent. Their experimental demonstrations are presented in Figure S4 (Supporting Information). This material flexibility allows TCW to operate on rigid, flexible, thick, or even curved surfaces – scenarios where EWOD often fails due to its strict lithographic requirements and dielectric thickness constraints. This flexibility allows customization of TCW‐based DMF systems to suit application‐specific needs such as wearable devices, biosensors, and optical platforms.

Notably, substrate materials listed above are all ranked on the negative side of the triboelectric series (see Figure S5, Supporting Information), enhancing compatibility with PTFE actuators. In contrast, materials such as glass, paper, stainless steel, and copper, which are ranked with positive triboelectric polarity, showed limited TCW performance when paired with negatively charged PTFE actuators. This is attributed to surface charge neutralization caused by positive‐polarity materials.

- User interactivity and reconfigurability

Unlike EWOD, where droplet motion is confined to pre‐defined electrode paths, TCW allows droplets to be guided freely in any direction. This makes the platform well‐suited for dynamic, reconfigurable fluidic applications. The ease of actuation, lack of electrical infrastructure, and intuitive fingertip control open new possibilities for point‐of‐care diagnostics, mobile testing platforms, and educational tools in under‐resourced environments.

- Enhanced safety and electrochemical stability

By avoiding direct voltage application, TCW eliminates common EWOD issues such as dielectric breakdown, electrolysis, and gas bubble formation. This improves both device reliability and safety, especially when handling biological fluids or salt‐containing solutions, which are prone to degradation under electric stress.

To clarify TCW's capabilities, various aspects of TCW are compared to EWOD in **Table** **1**. These attributes establish TCW as a robust, flexible, and user‐friendly alternative to traditional EWOD systems. With the ability to perform all core droplet operations using nothing more than tribo‐charged materials, TCW establishes a new paradigm of electric‐free microfluidic control. Its unique advantages ‒ elimination of electronics, insensitivity to substrate geometry, compatibility with flexible materials, and intuitive user operation ‒ position TCW as a powerful platform for next‐generation lab‐on‐a‐chip devices, especially in diagnostics, environmental sensing, mobile laboratories, and educational tools.

**Table 1:** A Comparison Table Summarizing the Differences between EWOD and TCW.

Category	EWOD	TCW
**Actuation Source**	External electric voltage is applied to the electrodes	Tribo‐charged material generates an electrostatic field through contact electrification
**Power Requirement**	External power supply and control circuitry required	Completely electric‐free (no power supply or electronic circuits needed)
**Device Architecture**	Complex: requires pixelated electrodes, wiring	Simple: only needs a tribo‐charged actuator
**Saturation Phenomenon**	Occurs at high voltage, limiting further angle change	Occurs at charge density, limiting further angle change
**Bubble Formation / Electrolysis**	Common at high voltages due to dielectric breakdown or imperfections	No bubble formation observed; avoids electrolysis since there's no applied voltage
**Dielectric Thickness Effect**	Thinner dielectric enhances performance but risks breakdown	TCW is insensitive to dielectric thickness
**Material Flexibility**	Limited due to fabrication constraints	High material compatibility (PTFE, PMMA, PET, PDMS, etc.)
**Cost and Scalability**	High cost due to lithography, PCB, and wiring	Low‐cost, scalable, user‐friendly
**Droplet Operations Demonstrated**	Transportation, merging, mixing, splitting (needs programmed control)	Transportation, merging, droplet generation – all electric‐free, even via fingertip motion
**User Interaction**	Requires software/hardware interface	Enables direct, intuitive control (e.g., fingertip actuator)
**Automation**	Automated by programming the control circuit	Manual operation is necessary
**Multifunctional Integrability**	Integration with bio and chemical sensors has been demonstrated	Integration of bio and chemical sensors is possible

## 5 Conclusion

This study establishes TCW as a fundamentally new and electric‐free mechanism for active surface wettability modulation. Unlike direct voltage‐driven EWOD, TCW harnesses surface charges generated through contact electrification to induce electrostatic forces across a dielectric, modulation the droplet's wettability. By eliminating the need for external power supplies, patterned electrodes, wiring, and electrical circuitry, TCW allows for significantly simplifying device architecture while enhancing portability and reducing fabrication complexity.

We experimentally demonstrated that TCW achieves substantial and reversible contact angle modulation (Δ*θ* = 44.4°), driven solely by tribo‐charged actuators without any voltage inputs. This charge‐driven operation avoids the risks of dielectric breakdown and electrolysis inherent in high‐voltage EWOD systems. Key governing factors such as surface charge density and actuator size were shown to dominate TCW performance, while dielectric thickness was found to have minimal influence. These characteristics allow for greater material and geometric flexibility compared to EWOD systems, enabling TCW operation on a wide range of substrates, including thick, flexible, chemically inert, and curved surfaces. The versatility of TCW was further validated through demonstrations of all essential DMF operations, including droplet transport, merging, and generation from a reservoir, executed entirely without any electronic components. Notably, continuous droplet transportation was achieved at speeds up to 40 mm s^−1^ on a single PTFE surface, even across non‐planar terrains. We further demonstrated fingertip‐driven droplet manipulation, highlighting TCW's unique potential for user‐interactive, low‐cost, and intuitive microfluidic platforms.

The successful realization of TCW not only advances scientific understanding of a new wettability control mechanism but also establishes a new class of electric‐free DMF platforms. This paradigm shift toward electric‐free operation paves the way for the development of low‐cost, user‐friendly, energy‐efficient, and highly portable DMF technologies, particularly in resource‐limited settings. By circumventing the limitations of traditional EWOD architectures, TCW holds immense potential for applications in healthcare diagnostics, environmental sensing, biochemical assays, and lab‐on‐a‐chip technologies.

## 6 Experimental Section

### Materials

PTFE films with various thicknesses, PTFE rods, and Nylon board were purchased from McMaster.

### Atmospheric Condition

All experiments were carried out under an average relative humidity of 60% at a room temperature setting ≈22 °C.

### Actuator's Surface Charge Density

A surface DC voltmeter (SVM2, AlphaLab Inc.) was used to measure the surface charge density of PTFE actuators. Charge density value can be measured up to hundredths of µC/m^2^. Before contact electrification, PTFE rods and Nylon board were both made free of any pre‐existing charges using an anti‐static gun (Zerostat 3, Milty). Complete removal of charges was confirmed using the surface voltmeter, ensuring that the surface voltage reading was negligibly below 5V, which corresponds to about *Q* = 9 pC. A PTFE rod was then rubbed against a stationary nylon board, where a frictional normal force was varied to achieve a large range of surface charge density. After rubbing, the surface charge density of PTFE actuators was calculated from the surface voltage measured by a surface voltmeter.

### Optical Setup

A charge‐coupled device camera (Kiralux, Thorlabs) was used to take front‐view images of droplet actuation during the wettability study and videos for the droplet manipulation study. Backlight and white screen were additionally used to obtain clear droplet images.

### Contact Angle Measurement

A 30 uL water droplet was loaded on the PTFE film surface. A tribo‐charged actuator was placed below the droplet to induce its wettability change. Using the ImageJ software, its front‐view images were analyzed to obtain contact angle data. Each data point in the experimental study represents the average value of the droplet's left and right contact angles at a given surface charge density in a single measurement. In experimental validation of TCW modulation, individual experimental data points were all plotted.

### Simulation Section

Actuator size (*D*_A_ = 3, 6, 12, and 24 mm) was variously set to follow the experimental conditions. A droplet was modeled as a hemisphere with its base diameter *D*_D_ = 3 mm for simplification and generalization of TCW on a hydrophobic surface. To study the material‐related effects, the thickness of a dielectric layer was variously selected as *t* = 25, 127, and 228 µm, and its relative permittivity was set to be *ε*_r_ = 2 to match with the experimental conditions. Surface charges on actuators were assumed to be uniformly distributed on its surface. The droplet was given the ground condition. All simulations were used an Extra Fine mesh, which was selected after a mesh convergence test. The voltage drop data at the droplet edges were extracted from COMSOL simulations. The *η* value was calculated using Equation (2) to assess the material‐related effects on the wettability modulation in TCW.

## Conflict of Interest

The authors declare no conflict of interest.

## Supporting information



Supporting Information

Supplemental Movie 1

Supplemental Movie 2

## Data Availability

The data that support the findings of this study are available from the corresponding author upon reasonable request.
