# Supplementary material for: Tribo‐Charge Induced Wetting (TCW): A New Wettability Control Mechanism for Electric‐Free Droplet Manipulation
Source: Adv Sci (Weinh). 2025 Oct 29;13(1):e11863. doi: 10.1002/advs.202511863 (PMC12767006; doi:10.1002/advs.202511863)
Supplement: Supplementary file 1 — Supporting Information [file ADVS-13-e11863-s003.pdf]

Supplementary Information

Title: Tribo-Charge induced Wetting (TCW): A New Wettability Control Mechanism for Electric-Free Droplet Manipulation

Authors: *Yeonwoo Lee and Sung-Yong Park**

1. Actuator's Charge Stability

The long-term stability of tribo-charged actuators is critical for ensuring reliable and repeatable TCW operation. Surface charge dissipation occurs through several mechanisms, including air breakdown [1, 2], surface conduction [3, 4] and thermionic emission [5]. Among these, air breakdown is recognized as the dominant discharge pathway under ambient conditions and has a direct impact on TCW performance [6]. Air breakdown is triggered when the local electric field exceeds the ionization threshold of surrounding air molecules. Under normal atmospheric conditions, this phenomenon occurs at a surface charge density of approximately $\sigma = 27$ μC/m^2 [7]. Beyond this threshold, strong localized electric fields induce air ionization, rendering nearby air regions partially conductive. This, in turn, accelerates charge leakage from the actuator surface, leading to performance degradation.

Figure S1 presents the time-dependent charge retention of PTFE actuators with different initial charge densities. The actuators were charged via frictional contact with a nylon plate, with varying normal loads applied to control the initial charge levels. Surface charge density was monitored over a 10-minute period under ambient conditions. At high charge densities, significant discharge was observed. For example, an actuator initially charged to $\sigma = -55.35$ μC/m² experienced a drop to − 45.37 μC/m², corresponding to an 18.1% charge loss. Similarly, an actuator starting at $\sigma = -41.25$ μC/m² decayed to − 34.99 μC/m², a loss of 15.3%. These results confirm that exceeding the air breakdown threshold leads to rapid charge dissipation. At sub-threshold charge densities, discharging was minimal. Actuators with initial charges of $\sigma = -21.33$ μC/m² and $\sigma = -9.11$ μC/m² retained 96.7% of their charge after 10 minutes, with final values of $\sigma = -20.60$ μC/m² and − 8.80 μC/m², respectively. These low discharge rates indicate excellent temporal stability within the practical operating range.

Importantly, most TCW-induced contact angle modulation occurs below the air breakdown threshold. Higher charge densities beyond $\sigma \approx 27$ μC/m² yield diminishing returns due to angle saturation, and do not contribute to further modulation. Therefore, for the typical working range employed in TCW applications, surface charges remain stable over extended periods, supporting consistent and reliable droplet actuation.

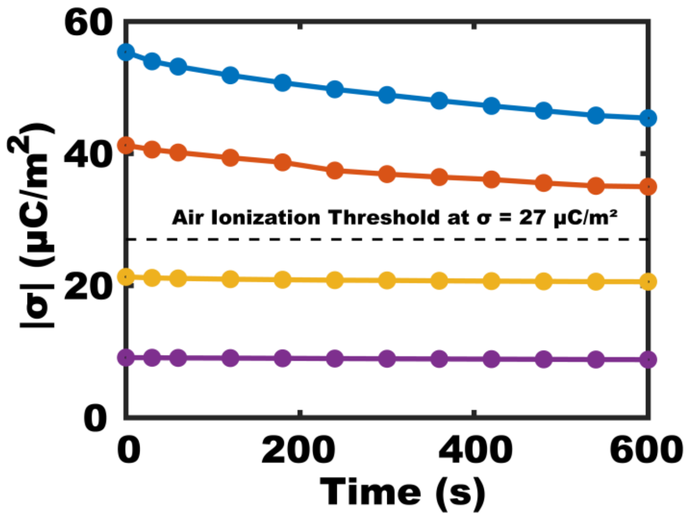

Figure S1. Measurement of surface charge density of the actuators over time. An initial charge density of the actuators was variously tuned by adjusting a frictional normal force between them. Their charge density was monitored using the surface voltmeter for a duration of 10 minutes to investigate the charge dissipation phenomena.

2. Additional Simulation Results on Thickness Effect Across All Actuator Sizes

To further validate the thickness-independence of TCW-based wettability control, we conducted extensive simulations analyzing the influence of dielectric layer thickness across a range of actuator sizes. **Figure S2** presents the variation of the dimensionless electrowetting number (η) as a function of dielectric thickness for normalized actuator diameters of (a) D^* = 1, (b) D^* = 2, (c) D^* = 4, and (d) D^* = 8, where D^* denotes the actuator size normalized to the droplet diameter. Across all configurations, η exhibits a plateau behavior below the contact angle saturation limit, confirming that TCW performance remains effectively independent of dielectric thickness within the practical working range. We define η_{limit} as the saturation threshold beyond which increases in charge density yield notably reduced contact angle change. These limits, determined experimentally for each actuator size, are as follows: (a) $\eta_{\text{limit}} = 0.17$ (corresponding to $\sigma = -$ 31.0 μC/m^2) for D^* = 1, (b) $\eta_{\text{limit}} = 0.31$ (corresponding to $\sigma = -$ 22.4 μC/m^2) for D^* = 2, (c) $\eta_{\text{limit}} = 0.43$ (corresponding to $\sigma = -$ 6.3 μC/m^2) for D^* = 4, and (d) $\eta_{\text{limit}} = 0.48$ (corresponding to $\sigma = -$ 1.7 μC/m^2) for D^* = 8, respectively.

The shaded green regions in **Figure S2** represent the practical operating range, capped by η_{limit} for each actuator size. Within this regime, dielectric thickness has minimal influence on η, underscoring TCW's robustness and design flexibility. As actuator size increases, η_{limit} also increases, enabling more effective contact angle modulation at lower charge densities. For instance, an actuator at D^* = 1 requires a high charge density ($\sigma = -$ 31.0 μC/m^2) to reach $\eta_{\text{limit}} = 0.17$, while an actuator at D^* = 8 achieves superior modulation ($\eta_{\text{limit}} = 0.48$) with a much lower charge density ($\sigma = -$ 1.7 μC/m^2). This relationship emphasizes the strong geometric dependency of TCW and highlights the advantage of using larger actuators for efficient operation within low-to-moderate charge densities.

These simulation results herein confirm that TCW's thickness insensitivity is preserved across all actuator sizes, as long as operation remains within the saturation-free regime. This property offers substantial design flexibility, allowing TCW systems to be implemented on a wide variety of dielectric substrates − including thick, flexible, and low-cost films − without sacrificing actuation performance.

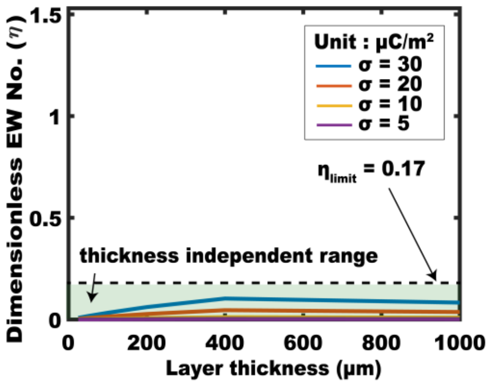

(a)

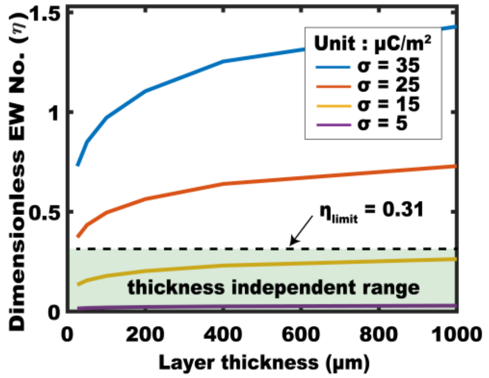

(b)

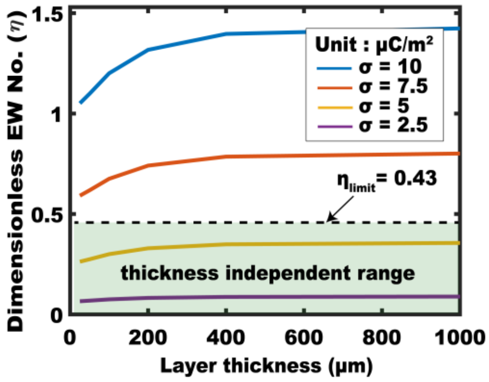

(c)

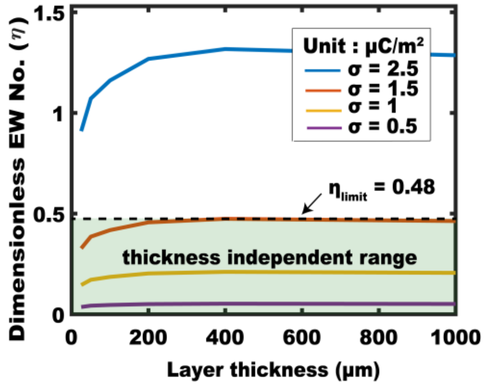

(d)

Figure S2. The dimensionless EW number (*η*) presented as a function of the dielectric thickness for (a) $D* = 1$, (b) $D* = 2$, (c) $D* = 4$, and (d) $D* = 8$.

3. Asymmetric Wetting for Droplet Transportation

Fig. 9(A) of the manuscript shows the video snapshots of continuous droplet transport as a tribo-charged actuator beneath it swipes from left to right. From the same video, we can clearly see the droplet's wetting behavior relying on the actuator's position relative to the droplet, as shown in **Figure. S3**: (a) At an initial state when the actuator is far away from the droplet, the droplet sits on a hydrophobic surface with the initial contact angles around $\theta_L \approx \theta_R \approx 121°$, (b) When the actuator moving to the right reaches the droplet center, it becomes wetted symmetrically, where the left and right contact angle is almost the same at $\theta_L = 116°$ and $\theta_R = 116.6°$, and (c) As the actuator continues to move to the right, the wettability change is much greater on the right side of the droplet with the contact angle of $\theta_R = 69.6°$, as compared to the left side at $\theta_L = 102.4°$. This imbalance in surface tension creates a surface energy gradient from left to right side of the droplet, driving the droplet to the right as mentioned in Section 4 of the manuscript. **Figure. S4** shows the simulation results for asymmetric actuation where D = 6 mm actuator charged at $\sigma = -40\ \mu C/m^2$ is displaced by 3 mm along the x-axis from the center (x = 0) beneath the 3 mm hemispherical droplet. In contrast to symmetric actuation simulated in Fig. 5 of the manuscript with voltage drop of 439V at both left and right droplet edges, asymmetric actuation results in V_L = 191V and V_R = 680V. Based on the Young-Lippmann Eq. (1) in the manuscript, a larger voltage drop at the right side creates more contact angle modification than the one at the left side. This charge-driven asymmetric wetting control enables the surface tension gradient as characterized by a smaller (i.e., more wetted) advancing angle (θ_{adv}) at the energized site by the actuator than the receding angle (θ_{rec}) at the left side. This asymmetry generates an internal pressure gradient inside the droplet, driving it toward the actuator placed.

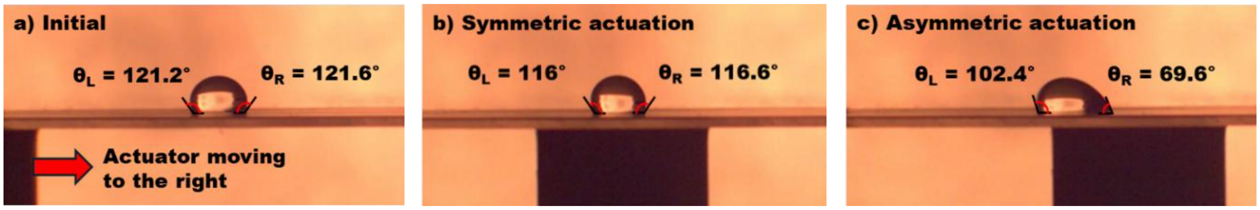

Figure. S3 Experimental demonstration of TCW-driven droplet transportation as shown in Fig. 9(A) of the revised manuscript. (a) At an initial state when the actuator is far away from the droplet, the contact angles of the droplet are observed as $\theta_L \approx \theta_R \approx 121°$. (b) When the actuator reaches the droplet center, a droplet is symmetrically fomed with the reduced contact angle at $\theta_L \approx \theta_R \approx 116°$. (c) As the actuator continues to move to the right, the wettability is largely modified on the right side of the droplet at $\theta_R = 69.6°$, as compared to the left side at $\theta_L = 102.4°$. This asymmetric angle change generates an internal pressure gradient, leading to droplet transporation as following the actuator.

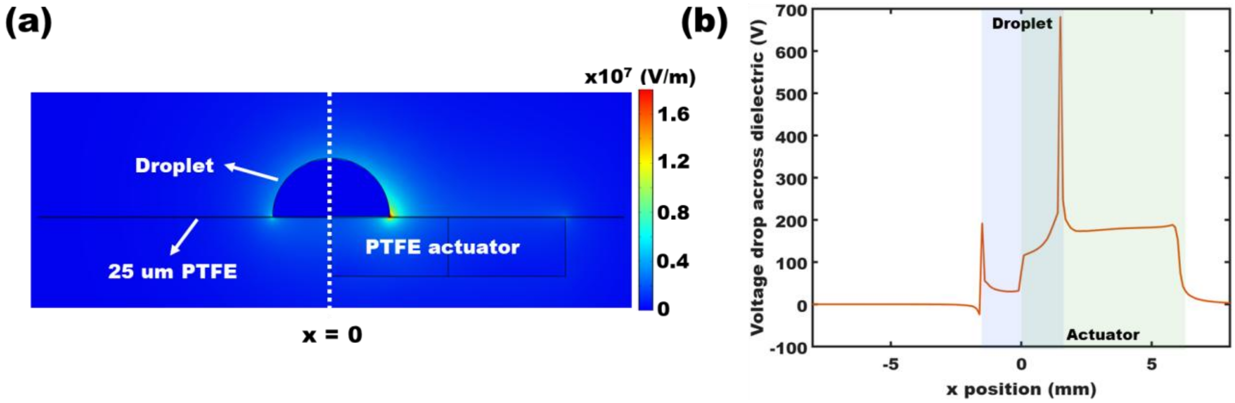

Figure. S4 (a) 3D numerical simulation of electric field distribution created by a circular actuator in 6 mm diameter charged at 40 μC/m^2 positioned below a 3 mm hemispherical droplet sitting on the surface of 25 μm PTFE film **(b) The voltage drop profile across the dielectric layer** are exactracted from the simulations and plotted along the lateral cross-sectional position (x-axis). An asymmetic distribution in voltage profiles is created when an actuator is asymmetically positined relative to the droplet.

4. Material Compatibility and Flexibility

To assess the platform's versatility, we experimentally demonstrated droplet transportation across a wide range of substrates. A 30 μL water droplet was deposited onto a 90 μm thick PTFE film, which served as the hydrophobic dielectric layer. This film was gently laminated onto various substrates, including PMMA (poly(methyl methacrylate)), PTFE (polytetrafluoroethylene), PET (poly(ethylene terephthalate)), PC (polycarbonate), PE (polyethylene), PP (polypropylene), PDMS (polydimethylsiloxane), Kapton tape, Parafilm tape, glass, Kraft paper, A4 paper, stainless steel and copper.

Figure S5 presents representative video snapshots of successful droplet transport on the PTFE-coated substrate of (a) 600 μm thick PDMS, (b) 1 mm PET, (c) 130 μm Parafilm, and (d) 60 μm Kapton, using a D^* = 4 actuator at surface charge densities at σ = − 29.84 μC/m^2, − 18.28 μC/m^2, − 30.01 μC/m^2, and − 31.61 μC/m^2, respectively. These results confirm that TCW functionality is preserved across diverse substrates, many of which are flexible, chemically inert, biocompatible, optically transparent, or even curved surfaces – scenarios where EWOD often fails due to its strict lithographic requirements and dielectric thickness constraints. This material flexibility and compatibility enable the customization of TCW-based digital microfluidic (DMF) platforms for a wide array of application-specific needs, including wearable devices, biomedical sensors, and transparent optofluidic systems.

Importantly, all substrates that supported successful droplet transport are positioned on the negative side of the triboelectric series (highlighted in green in **Figure S6**). This alignment enhances compatibility with negatively charged PTFE actuators, allowing effective electrostatic interaction without significant charge neutralization. In contrast, droplet transport failed on substrates such as glass, Kraft paper, A4 paper, stainless steel, and copper. These materials are ranked on the positive side of the triboelectric series (highlighted in red in **Figure S6**) and tend to accumulate positive surface charges upon interaction with the negatively charged PTFE actuators. This polarity mismatch results in partial charge neutralization, thereby suppressing the electrostatic forces required for TCW-based droplet actuation.

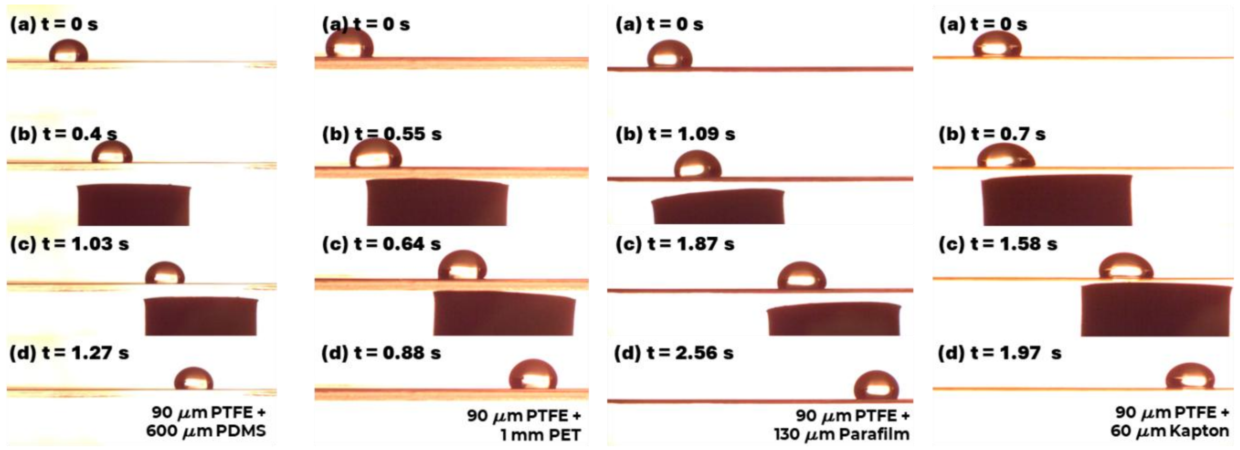

(A) (B) (C) (D)

Figure S5. Experimental demonstration of the TCW's capabilities for droplet transportation on a variety of substrate materials, including (A) 600 μm PDMS (B) 1 mm thick PET (C) 130 μm Parafilm tape and (D) 60 μm Kapton tape. The same actuator size at D^* = 4 was used for all experiments.

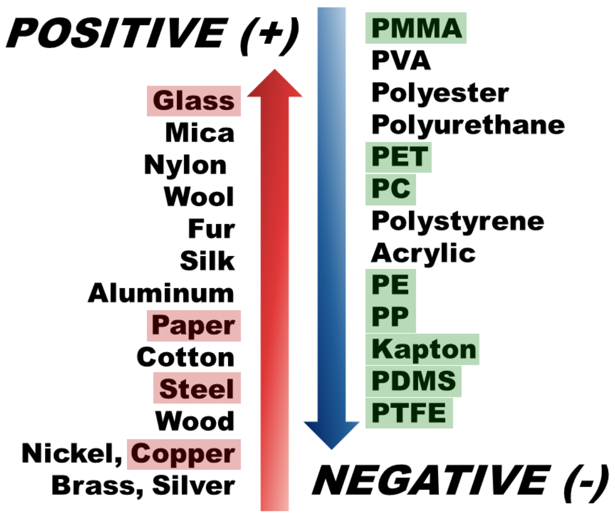

Figure S6. Triboelectric series with working (labeled green) and failed (labeled red) substrate material for TCW-driven droplet transportation.

References:

[1] Y. Zi, C. Wu, W. Ding, and Z. L. Wang, "Maximized Effective Energy Output of Contact-Separation-Triggered Triboelectric Nanogenerators as Limited by Air Breakdown," *Advanced Functional Materials,* vol. 27, no. 24, p. 1700049, 2017

[2] Z. Su, M. Han, X. Cheng, H. Chen, X. Chen, and H. Zhang, "Asymmetrical Triboelectric Nanogenerator with Controllable Direct Electrostatic Discharge," *Advanced Functional Materials,* vol. 26, no. 30, pp. 5524-5533, 2016

[3] M. Navarro-Rodriguez, E. Palacios-Lidon, and A. M. Somoza, "The surface charge decay: A theoretical and experimental analysis," *Applied Surface Science,* vol. 610, p. 155437, 2023

[4] H. Guan, X. Chen, H. Du, A. Paramane, and H. Zhou, "Mechanisms of surface charge dissipation of silicone rubber enhanced by dielectric barrier discharge plasma treatments," *Journal of Applied Physics,* vol. 126, no. 9, p. 093301, 2019

[5] X. Xia and Y. Zi, "Heat-Excitation-Based Triboelectric Charge Promotion Strategy," *Advanced Science,* vol. 11, no. 41, p. 2404489, 2024

[6] Y. Liu *et al.*, "Quantifying contact status and the air-breakdown model of charge-excitation triboelectric nanogenerators to maximize charge density," *Nature Communications,* vol. 11, no. 1, p. 1599, 2020

[7] C. K. Ao *et al.*, "Balancing charge dissipation and generation: mechanisms and strategies for achieving steady-state charge of contact electrification at interfaces of matter," *Journal of Materials Chemistry A,* 10.1039/D2TA03232E vol. 10, no. 37, pp. 19572-19605, 2022
